# Treatment of Klippel-Feil syndrome with symptomatic atlantoaxial instability in a 7-year-old boy: A case report

**DOI:** 10.1007/s00132-024-04537-z

**Published:** 2024-08-08

**Authors:** W. Pepke, T. Renkawitz, S. Hemmer

**Affiliations:** Department of Orthopaedics, Heidelberg University Hospital, Schlierbacher Landstr. 200a, 69118 Heidelberg, Germany

**Keywords:** Klippel-Feil Syndrome, Atlanto-Axial Instability, Myelopathy, Deformity, Surgical navigation, Klippel-Feil Syndrom, Atlantoaxiale Instabilität, Myelopathie, Deformität, Intraoperative Navigation

## Abstract

Klippel-Feil syndrome (KFS) is a congenital deformity of the cervical spine. Clinical symptoms of KFS are reduced range of motion, short neck and low hairline. In adult KFS patients the deformity can lead to adjacent segmental instability with spinal canal stenosis, radiculopathy and myelopathy. This article reports about the diagnostics and treatment management of juvenile KFS patient with myelopathy due to instability of the C1/C2 segment, subsequent stenosis through the posterior arch of C1 and symptomatic myelopathy. This 7‑year-old boy could be successfully treated with C1 decompression and computer tomography (CT) guided C1/C2 stabilization with pedicle screws under intraoperative neuromonitoring.

## Abstract (de)

Das Klippel-Feil Syndrom (KFS) gehört zu den kongenitalen Deformitäten der Halswirbelsäule (HWS). Klinisch manifestiert sich diese Erkrankung durch reduzierte Beweglichkeit der HWS, kurzen Hals und tiefen Haaransatz. Die KFS basierte Instabilität der Segmente außerhalb der kongenitalen Fusion mit konsekutiver Stenose, Radikulopathie und gar Myelopathie wird normalerweise im adulten Alter diagnostiziert. Dieser Artikel berichtet über die durchgeführte Diagnostik und die chirurgische Therapie eines 7‑Jährigen KFS-Patienten mit symptomatischer Myelopathie als Folge der C1/C2-Instabilität und einer dadurch bedingten hochgradigen zervikalen Stenose. Der Patient wurde erfolgreich mittels Dekompression in Höhe C1 und einer computertomographisch (CT-) navigierten Stabilisierung C1/C2 mittels Pedikelschrauben unter permanenter intraoperativer Neuromonitoring-Kontrolle chirurgisch versorgt.

## Introduction

Klippel-Feil syndrome (KFS), also known as cervical vertebral fusion syndrome [1], is a rare deformity characterized by the congenital fusion of two or more cervical vertebrae [1] due to faulty segmentation and resegmentation along the developing embryonal axis during the first 3–8 weeks of gestation [2]. The segmental fusion results in a limited range of motion and a short neck with a low hairline [3], often accompanied by an upward translation of the upper thorax [4]. The KFS was first reported independently by Maurice Klippel and André Feil in 1912 (see Van Kerckhoven and Fabry [3]). The syndrome was classified into three categories:

- Type I: fusion of C2 and C3 with occipitalization of the atlas,
- Type II: long fusion below C2 with abnormal occipitocervical junction,
- Type III: a single open interspace between two fused segments.

The estimated incidence of KFS is approximately 1 in 42,000 newborns [5]. The actual prevalence is believed to be higher due to missed diagnoses caused by the heterogeneity in phenotypic expression [3]. Treatment for KFS is symptomatic and may include surgery to relieve cervical or craniocervical instability and spinal cord constriction in rare cases. The KFS can cause radiculopathy or myelopathy due to stenosis and instability [6, 7], with cervical myelopathy being the most debilitating complication, usually caused by spinal canal stenosis at the C1 level [8]. Surgery is indicated for KFS patients with recurrent and progressive neurological symptoms, generally involving C1 decompression with craniocervical fixation [6, 7].

This article reports on a KFS patient with myelopathy due to atlantoaxial instability and the performance of C1–C2 dorsal stabilization with C1 decompression. The necessity of craniocervical fixation in KFS patients with atlantoaxial instability remains unclear. As the upper cervical spine (segments C0/C1 and C1/C2) contributes up to 70% of the cervical range of motion (ROM), craniocervical stabilization significantly restricts the cervical spine ROM. It is also uncertain if successful outcomes can be achieved with C1 decompression and monosegmental stabilization (C1/C2) in these patients.

## Clinical findings

A 7-year-old boy was presented to this department with mild and progressive neurological symptoms over 6 months. He reported mild weakness in the lower and upper extremities while walking or running and dizziness that prevented him from attending school sports for 3 months. Despite patterns consistent with KFS, he had no other spine-related symptoms like neck pain or cervicobrachial neuralgia.

**Fig. 1:**
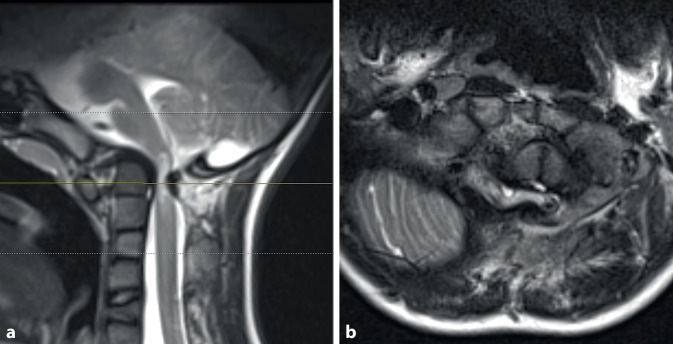
Magnetic resonance images in sagittal (**a**) and axial (**b**) planes showing the severity of cervical stenosis with myelopathy at C1 level

**Fig. 2:**
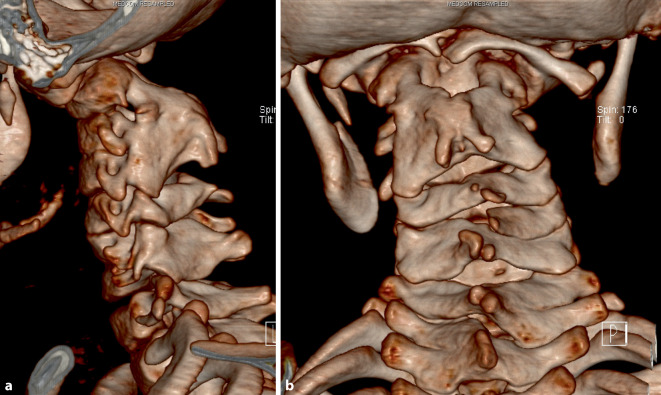
3D-CT reconstruction of cervical spine showing the severity of the deformity (**a** lateral view, **b** dorsal view)

**Fig. 3:**
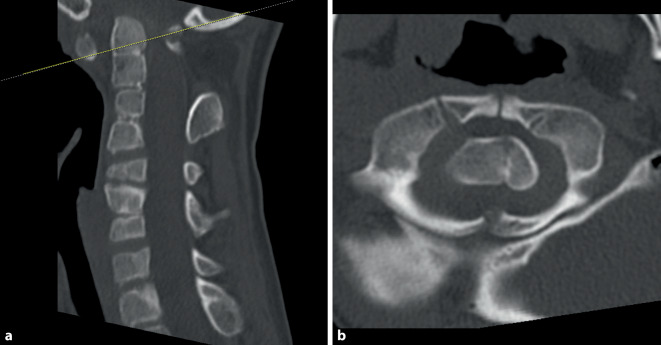
Computed tomography scans in sagittal (**a**) and axial (**b**) planes showing the severity of cervical stenosis caused by posterior arch of C1 and C1/C2 instability

Neurological examination revealed no significant paresis or vegetative dysfunction but a locomotor system disorder characterized by poor coordination with an unsteady stance and gait (stance and gait ataxia) was noted, leading to a suspected diagnosis of cervical myelopathy. Cervical spine radiographs showed abnormally configured cervical vertebrae with multilevel fusion in the upper segments. The atlantodental interval (ADI) was 5 mm in the lateral view. Magnetic resonance imaging (MRI) revealed severe spinal canal stenosis at the C1 level with a 4 mm distance between the odontoid process and posterior arch of C1, indicating severe narrowing of the upper cervical myelon at C1 (Fig. 1). High signal intensity cord lesions observed on T2-weighted images were consistent with myelomalacia and a 16 mm longitudinal myelopathy. Computed tomography (CT) confirmed the suspected deformity with multilevel segmental fusion of the upper cervical spine, according to KFS type II (Figs. 2 and 3). An excessive kyphotic change at the C1/C2 segment resulted in stenosis between the odontoid process and C1 posterior arch, and basilar invagination was diagnosed. Radiographs of the cervical spine in flexion and extension revealed instability at the C1/C2 level, with an ADI of 7 mm in flexion and 4 mm in extension. Contrast agent injection precisely showed the position of the vertebral arteries on both sides.

## Diagnosis

The patient was diagnosed with severe cervical myelopathy with upper cervical stenosis due to C1/C2 instability associated with KFS type II.

## Management

The pathology was compounded by severe cervical stenosis predominantly caused by the posterior arch of C1, accompanied by instability of the C1/C2 segment. To adequately address both elements of the pathology, it was decided to perform C1/C2 stabilization with extensive decompression of the C1 posterior arch. The surgery was planned with intraoperative neuromonitoring and navigation. The initial CT scan for diagnostics was performed per protocol requirements for the navigation system, avoiding additional radiation exposure for this 7‑year-old patient.

**Fig. 4:**
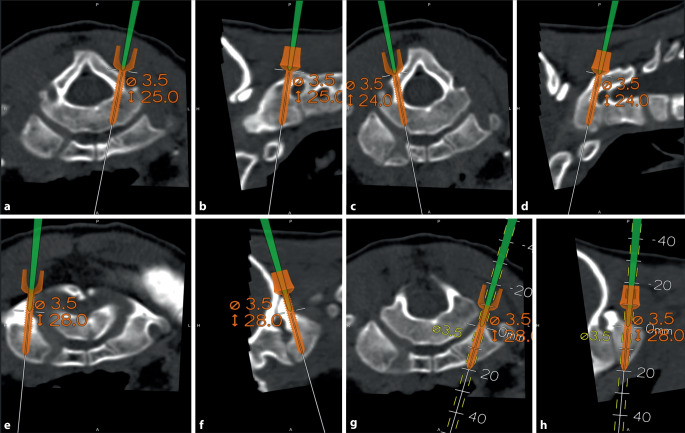
Intraoperative planning of screws length and navigation for screws implanting. **a** axial view, **b** sagittal view, **c** axial view, **d** sagittal viewe, **e** axial view, **f** sagittal view, **g** axial view, **h** sagittal view

**Fig. 5:**
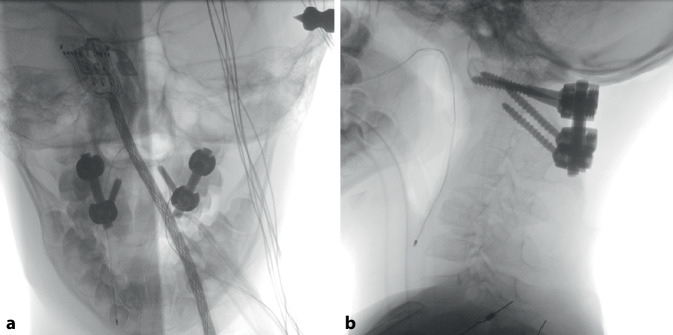
Intraoperative radiographs in lateral (**a**) and anteroposterior (**b**) planes after stabilization procedure. **a** anteroposterior view and **b** lateral view

After fiberoptic intubation to avoid dorsal neck extension during the procedure, the patient was anesthetized using total intravenous anesthesia (TIVA) without relaxants to enable appropriate neurophysiological monitoring. In the supine position, initial neural deduction of the four extremities was performed to establish a baseline for SSEP, MEP, and EMG during the surgical procedure. The patient was then moved to a prone position and his head was fixed in a Mayfield skull clamp. Further neural deduction was performed after repositioning of the C1/C2 segment. The planned surgery proceeded under continuous neural monitoring, with drilling, screw length planning, and pedicle screw insertion guided by a navigation system based on the preoperative CT scan (Figs. 4 and 5). An intraoperative low-dose CT scan confirmed the appropriate position of the pedicle screws. Extensive decompression through resection of the posterior arch of C1 was performed without pathological changes in neural monitoring. Postoperatively, the patient showed no signs of paraparesis.

The patient was mobilized to a standing position immediately after surgery, and the proprioception in the legs improved. Physical therapy and active exercises were prescribed and the patient was discharged 10 days postoperatively with a normal gait and muscle power (grade 5/5) in arms and legs. The gait ataxia further improved within 2 months after surgery. No postoperative complications were observed within 3 months, and further ambulatory controls are planned.

## Discussion

The symptoms experienced by KFS patients depend primarily on the severity of the deformity. In patients with less severe involvement cervical anomalies are often discovered incidentally during radiography examinations and can lead to normal lives without significant restrictions. Therefore, KFS management is predominantly conservative; however, about 35% of adult KFS patients experience symptoms related to the syndrome, such as neck pain, radiculopathy, and myelopathy [9]. Spinal canal stenosis can result from congenital deformity or degenerative processes above, below, or at the level of fused segments, causing myelon compression and myelopathy [7]. Degenerative processes in KFS patients include bulging discs, ligament hypertrophy, bony spurs, spinal canal stenosis, and instability, typically occurring at the mobile segment adjacent to the fused vertebrae [10]. Atlantoaxial instability is recognized by an ADI exceeding 4 mm [11] or a difference exceeding 3.5 mm in functional radiographies (flexion and extension) of the cervical spine in the lateral view [12]. In our patient, in addition to typical KFS segmental fusions, there was an anterior translation of C1 due to the hypermobility of the C1/C2 segment, associated with severe myelopathy caused by stenosis due to the C1 posterior arch.

Cervical pedicle screw insertion is challenging due to the small diameter of the pedicles and the closeness of neurovascular elements. A CT scan guided navigation improves the accuracy of pedicle screw placement [13, 14], particularly in the presence of deformity in children [13, 15]. Therefore, enhancement of safety and shorter fusion levels can be achieved [13].

## Conclusion

Patients with KFS can develop myelopathy due to segmental instability and stenosis between the odontoid process and posterior arch of C1. Instability can be aggravated by excessive range of motion of the C1/C2 segment due to segmental fusion of lower segments. This report aims to highlight the potential consequences of KFS and presents a treatment concept for coexistent instability in the C1/C2 segment with stenosis and myelopathy.

### Key points.

KFS may cause upper cervical spine instability, leading to stenosis and myelopathy in juvenile patients.

- Upon recognition of KFS, further diagnostics should be performed to exclude segmental instability.
- Surgical treatment of KFS with segmental instability and myelopathy is complex and requires intraoperative neuromonitoring and CT-guided navigation for patient safety.
- Treatment of these patients should be performed in spinal surgery centers equipped with intraoperative neuromonitoring and navigation.

## Data Availability

The datasets used and/or analyzed during the current study are available from the corresponding author on reasonable request.

## Abbreviations

ADI: Atlantodental instability
CT: Computer tomography
EMG: Electromyography
KFS: Klippel-Feil syndrome
MEP: Motor evoked potentials
MRI: Magnetic resonance imaging
ROM: Range of motion
SSEP: Somatosensory evoked potentials

## Declarations

For this article no studies with human participants or animals were performed by any of the authors. All studies mentioned were in accordance with the ethical standards indicated in each case. For images or other information within the manuscript which identify the patient, consent was obtained from them and/or their legal guardians.
